# Carbon Materials Advancing Microorganisms in Driving Soil Organic Carbon Regulation

**DOI:** 10.34133/2022/9857374

**Published:** 2022-01-12

**Authors:** Chunyu Tang, Fan Yang, Markus Antonietti

**Affiliations:** ^1^School of Water Conservancy and Civil Engineering, Northeast Agricultural University, Harbin 150030, China; ^2^Joint Laboratory of Northeast Agricultural University and Max Planck Institute of Colloids and Interfaces (NEAU-MPICI), Harbin 150030, China; ^3^Max Planck Institute of Colloids and Interfaces Department of Colloid Chemistry, 14476 Potsdam, Germany

## Abstract

Carbon emission from soil is not only one of the major sources of greenhouse gases but also threatens biological diversity, agricultural productivity, and food security. Regulation and control of the soil carbon pool are political practices in many countries around the globe. Carbon pool management in engineering sense is much bigger and beyond laws and monitoring, as it has to contain proactive elements to restore active carbon. Biogeochemistry teaches us that soil microorganisms are crucial to manage the carbon content effectively. Adding carbon materials to soil is thereby not directly sequestration, as interaction of appropriately designed materials with the soil microbiome can result in both: metabolization and thereby nonsustainable use of the added carbon, or—more favorably—a biological amplification of human efforts and sequestration of extra CO_2_ by microbial growth. We review here potential approaches to govern soil carbon, with a special focus set on the emerging practice of adding manufactured carbon materials to control soil carbon and its biological dynamics. Notably, research on so-called “biochar” is already relatively mature, while the role of artificial humic substance (A-HS) in microbial carbon sequestration is still in the developing stage. However, it is shown that the preparation and application of A-HS are large biological levers, as they directly interact with the environment and community building of the biological soil system. We believe that A-HS can play a central role in stabilizing carbon pools in soil.

## 1. Introduction

The “Greenhouse Effect,” induced e.g. by carbon dioxide (CO_2_) and methane (CH_4_), constantly sparks interest in carbon management and climate regulation, and a large body of literature has developed on that subject over the past decades [1–4].

CO_2_ is broadly discussed as the “greenhouse gas.” With the advent of the so-called *Anthropocene*, the amount of CO_2_ in the atmosphere by human activity rapidly increased, from 280 ppm before the industrial revolution to over 415.48 ppm in June, 2021 [5, 6]. CO and CH_4_ emissions also contribute notably to the greenhouse effect; although, they are minor compared to CO_2_ emissions [7]. Soil, as one of the important places to stock C, plays an often-overseen role in the global C cycle. The popular perception is that the burning of coal and fossil fuels is solely responsible for the climate change [6]. This is, however, only one of the involved phenomena. Soil is presumably the greatest active C pool of the geosphere with 2,344 Gt of organic carbon [8], and thereby, the C content within soil organic matter is more than three times higher than that the C content of the atmosphere or terrestrial vegetation, globally [9]. Moreover, soil is a huge C emitter, and the amount of CO_2_ emission (appropriately 60 pg/yr) from soil is ten times higher than that of fossil fuels [10], while its C flux is six to ten times the amount of CO_2_ emitted by human activities [11]. As a rough estimate, once the effluxes of C in soil are slightly larger than the influxes (say 1%), huge amounts of C will be liberated [12], here 10 times the amount of fossil C emissions [13]. Huge amounts of these effluxes from soil are caused by anthropogenic actions, e.g., altered land use and improper agriculture practices. The fluxes and the management of soil carbon, as the biggest C source and potential C-sink, have to receive more attention to address global warming [8, 13–15].

Carbon loss from soil does not only hold important ramifications for global climate change but also has massive effects on global food security [16, 17]. It was claimed that the world will face a severe food crisis unless food production is increased by 60~110% from 2005 to 2050 [16], while at the same time, arable soil fertility is constantly decreasing due to overfarmed land substantially losing soil organic matter (SOM) and the essential nutrients that adhere to SOM [18]. Previous investigations have revealed that SOM depletes for various reasons, e.g., soil erosion due to large-scale reclamation [17, 19], improper plowing, and excessive use of fertilizers [20–22], even a warming climate. Consequently, new techniques that enable to reenrich soil carbon have to become first priority in worldwide science to ease the food, and climate crisis that will occur in the future has already started.

To date, the investigations regarding the support of artificial carbonaceous substances, e.g., biochar or humic substance (HS), to soil C pool, are advancing [23]. Such chemical engineering processes can complement reasonable farming systems, such as no tilling, mulching, and crop rotation [18, 24, 25]. The resilience of artificial carbonaceous substances against metabolization is considered critical to building up stable C pools in soil [26–29]. As a base of a possible biological nonlinear amplification of carbon sequestration, biochar and HS also increase/support plant growth inducing intensified photosynthesis which sequesters more CO_2_ from atmosphere [26, 30]. Additionally, there is growing focus regarding the importance of artificial carbon materials, e.g., biochar and A-HS, supporting and reinforcing the growth, metabolism, and community structure of microbes in soil [31, 32]. Microbial C sequestration has, in our opinion, the greatest potential for carbon fixation because of the various and complementary autotrophic C sequestration schemes [33–37].

Overall, the main objects of the review are to analyze the feasibility of enhancing soil C sequestration via microbial communities and dissect the effect of artificial carbon materials on microecological management, with some focus on A-HS synthetized by our group [38].

## 2. Soil Carbon Flux Derived from Microorganisms

It is well known that microorganisms in soil both emit and sequester C. C emission from soil is in the form of CO_2_ in many cases, labeled as total soil respiration (*R*_*S*_), covering biological and nonbiological processes. *R*_*s*_ is the largest CO_2_ flux from soil to atmosphere and is constantly increasing over recent years [39], while microorganisms and their diversity have a vital role in soil respiration [40]. The production of methane (CH_4_) and its aerobic/anaerobic oxidation (CH_4_⟶CO_2_) is another path for soil microbes to participate in soil C emission [41].

Correspondingly, C sequestration refers to the transformation of atmosphere CO_2_ into soil organic matter. Microorganisms are also involved in C fixation through anabolism, besides emitting CO_2_ through respiration [34], and thereby start the soil C cycle [42–44]. Microbial necromass critically contributes to soil C flux and accumulates as SOM [45]. In the following paragraphs, the main five pathways of microorganism to participate in soil C circulation will be reviewed.

### 2.1. Microbial Involvement in Carbon Emissions

#### 2.1.1. Carbon Dioxide Emissions by Microbial Respiration

**Figure 1:**
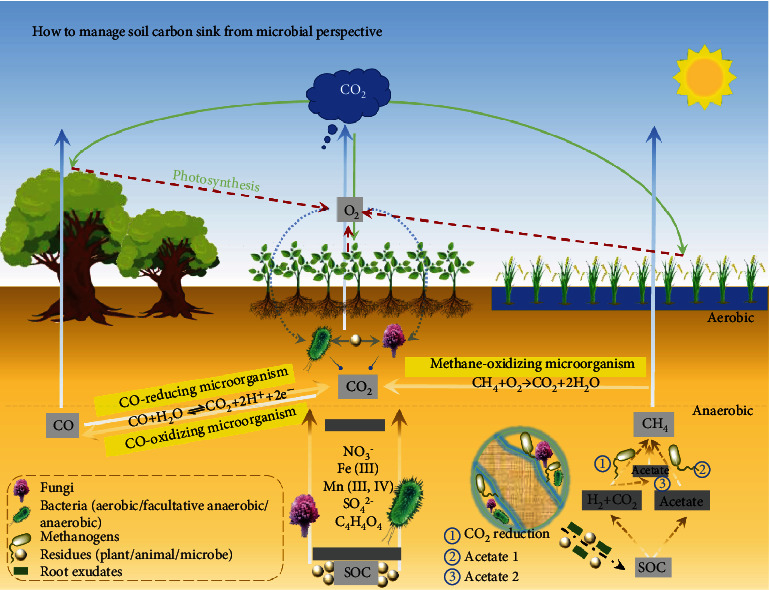
Schematic diagram of microbial involvement in carbon emissions.

The biological processes of soil respiration mainly contain microbial, root, and soil animal respiration [46, 47]. A large stock of investigations validates that microbial heterotrophic respiration (*R*_*H*_) decomposing soil organic matter (SOM) and plant debris [48] largely contribute to soil respiration [49, 50], under both aerobic and anaerobic conditions. The rate at which microorganisms oxidize and decompose organic matter in the presence of oxygen is unquestionably high. The role of anaerobic respiration on CO_2_ emission is also important; although, the flux of CO_2_ can be 10 times slower than that of aerobic respiration [51]. For example, Fe- (III-) reducing microorganisms are a typical model of anaerobic respiration. The microbes/microorganisms utilize Fe (III) as an electron acceptor, labeled as Fe respiration (Figure 1) [52, 53]. This process contributes to the mineralization of organic C on a scale of up to 44% in soils that suffer of consistent redox fluctuations [54]. Bond-Lamberty et.al [49, 55] revealed in a detailed and various insight that the critical rise of *R*_*H*_ is responsible for most of the CO_2_ flux. *R*_*H*_ is moreover sensitive to the rise of temperature, which starts a fatal cycle, i.e., excess soil C emission makes the climate warm [13], while a warming climate improves *R*_*H*_ [56]. As one example, the response of microbial respiration to warming climate was investigated by Nottingham et.al. [57] through burying warming rods in a tropical forest on Barro Colorado Island, Panama. Compared with soil at an ambient temperature, the overall soil profile was warmed by 4°C over two years, resulting in a 55% increase in CO_2_ emission originating from heterotrophic respiration. At the same time, rainfall, soil erosion, the amount of minerals, etc., are also considerably critical for *R*_*H*_ [13, 58–61].

#### 2.1.2. Methane/Carbon Monoxide Emissions

Methanogenesis is another anaerobic respiration path. Statistically, the emission of methane (CH_4_) has increased up to 1800 ppbv [62], which is approximately three times the value at the beginning of the industrial period. Methane (CH_4_), which is generated by methanogenes mainly existed in anoxic soil environments, e.g., paddy soils [63], is another major greenhouse gas [64, 65].

In general, soil methanogens produce CH_4_ along three pathways (Figure 1). The first one is called *CO_2_ reduction pathway*, and it contributes 10-30% to the CH_4_ production [66]. During this reaction, CO_2_ and H_2_ are produced by hydrolysis and fermentation, which is induced by either hydrolytic or fermentative microflora (syntrophs) and utilized by hydrogenotrophic methanogen to generate CH_4_ [37]. The second way is the acetate pathway 1 and is responsible for 70-90% of CH_4_ production [66]. In this process, acetate is produced by fermentation, and aceticlastic methanogens then generate CH_4_ [7]. The third way is the acetate pathway 2. Herein, acetate is produced by the reduction of CO_2_ via the acetyl-CoA pathway (acetogenesis) and then is again utilized by aceticlastic methanogens to generate CH_4_ [67]. 40% of the CH_4_ produced as such could be released into the atmosphere [7]. Alternatively, in a well-operating microbial system, the produced CH_4_ is used as an energy source and is back-oxidized to CO_2_ by methane-oxidizing microbes in a coupled aerobic environment [41, 63, 68] (Figure 1).

Carbon monoxide (CO), a minor, but relevant C-related gas emitting from soil [69], is delivered by the abiotic degradation (e.g., thermal degradation and photodegradation) of carbohydrates and lignin from plant litter [70, 71]. The incomplete chemical oxidation of organic C in soil at low oxygen partial pressures is an additional path of CO formation and release [72]. Biotic reduction of CO_2_ is another source of CO in soil, which is performed by anaerobic bacteria, e.g., sulfate-reducing bacteria [73] or methanogenic bacteria [74]. The progress of CO production by microbes is also displayed in Figure 1, and crossing the dotted line indicates that the process can occur under both aerobic and anaerobic conditions. At the same time, soil is also a CO sink from the gas phase and critically contributes to the CO consumption from the atmosphere [69, 75].

### 2.2. Microbial Carbon Sequestration

#### 2.2.1. Microbial Carbon Dioxide/Methane/Carbon Monoxide Sequestration

**Figure 2:**
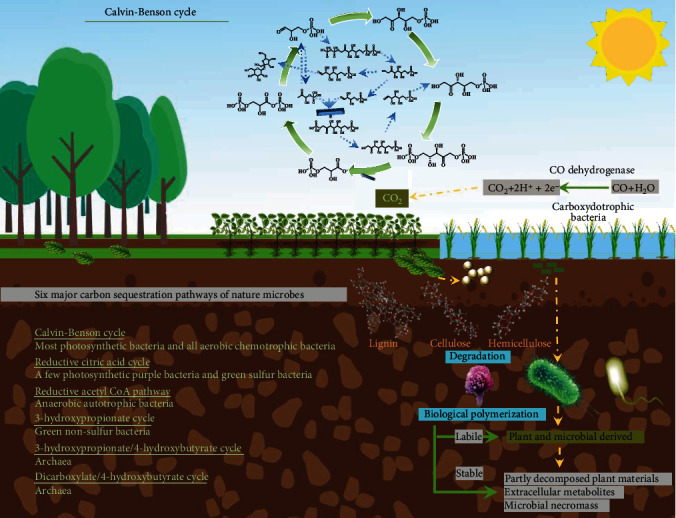
Schematic diagram of microbial involvement in carbon sequestration.

Six natural C sequestration pathways of microbes are commonly acknowledged, where the Calvin-Benson cycle is the most common C sequestration pathway of many prokaryotes [33, 76], e.g., cyanobacteria, purple bacteria, and aerobic chemoautotrophic bacteria (Figure 2). As reported, CO_2_ assimilation by autotrophs, that is the transformation of inorganic C to organic C, turns around 7 × 10^16^ g of C annually [34]. Lynn et al. [77] investigated autotrophic C sequestration for three different natural ecosystems (wetland, grassland, and forest) based on the ^14^C-labeling technology. The literature shows that microbial assimilation of ^14^C contributed 14.2%, 15.3%, and 20.2% to ^14^C-labeled SOC in wetland, grassland, and forest ecosystems, respectively. In addition to C sequestration by autotrophic microbes, some heterotrophic microbes also conduct C sequestration, for instance, by C-H Bond carboxylation [78], e.g., *Propionibacterium pentosaceum* [79], and *Bacterium coli* [80]. In these cases, both the substrates and energy originate from the decomposition of organic compounds.

CO is also a well-known energy source for the growth of microbes [81] (Figure 2). In detail, carboxydotrophic bacteria operate by oxidizing CO with H_2_O to CO_2_ and 2H^+^ +2e^−^ and depend on CO dehydrogenase (CODH) within the Calvin-Benson cycle [82]. Typically, *Pseudomonas carboxydovorans* [75], *Alphaproteobacteria* (Purple non-sulfur bacteria) [83], and several carboxydotrophic bacteria are capable to use CO directly, i.e., incorporating CO-C into the carboxyl group of acetate with acetyl-CoA synthases in the Wood-Ljungdahl pathway [84, 85].

#### 2.2.2. Microbial Necromass

A novel theory, proposed by some soil scientists [86, 87], is that the most persistent organic C in soils probably is not based on plant litter or its residues, but on C that passes first through microbial biomass (Figure 2). In 2019, Liang et al. [45] accurately designed a strategy for quantitating the contribution of microbe derived C to the topsoil organic C pool in temperate agricultural, grassland, and forest ecosystems. The approach was based on the integration of existing biomarker amino sugar data (1996~2018) within an ecological systems approach, elemental C-nitrogen stoichiometry, and biomarker scaling. One striking result was that microbial necromass contributed up to half of the SOC. Benner [86] strongly emphasized that the mechanism how heterotrophic microorganisms form a stable organic C pool depends on the resistance of microbial remnants products, e.g., hardly digestible biomolecules as lipopolysaccharides and hopanoids. Their unique structure helps them to resist the degradation in soils, and for instance, intact hopanoids were found being 2,500 million years old [88].

Complexes formed by microbial necromass and soil minerals, especially Fe minerals, are another important mode of soil organic carbon accumulation by involved microbial actions [89, 90]. Up to date, the complexation between soil organic carbon and soil minerals has been constantly investigated and is now considered as an effective form of stabilized carbon [91]. Microbial necromass was also in other work described as an important carbon source to bind with soil minerals [92]. The cell envelop comprised of lipid bilayers, proteins embedded into these bilayers, peptidoglycanes, and lipopolysaccharides or teichoic acids, is potentially stabilized by soil minerals depending on its macromolecular architecture, increasing its resistance to microbial degradation [93]. A long-term investigation was done by Wang et al. [94] regarding the stabilization of microbial necromass supporting the contribution of soil minerals to stabilize SOM derived from microbial necromass. In this analysis, the relative recovery of microbial necromass ^15^N, including bacteria, fungi, and actinobacteria, in mineral associated organic matter accounts for approximately 20%, directly supporting the viewpoint that mineral bonding stabilizes the soil carbon pool.

#### 2.2.3. Biologically Polymerizing Small Molecules to Form Humic Substance by Microbes

The pathway how microbes play their key role in the formation of soil HS is manifold, but contain (i) decomposition: animal and plant debris are broken down into small molecules by microbes, fungi, bacteria, etc., to fuel their life and (ii) transformation and synthesis: the easily digestible part is turned into microbial biomass and partly rearranged into larger and less digestible units in the microbes. Within this process, humic acid (HA), fulvic acid (FA), and humic matter (HM) are left after repeated partial metabolization of microbes [95]. HA and HS are relatively stable carbon carriers and increase the soil C pool (Figure 2).

## 3. Potential Regulation of Soil Carbon Pool

### 3.1. Abiologically Regulated Carbon

In recent years, in light of improving farming techniques to increase SOC, e.g., no till, mulching, and crop rotation [18], the addition of human-made artificial carbon materials (ACM) received considerable attention, due to the potential sustainability and stability of the sequestered C [26, 96]. This represented indeed the first carbon-negative countermeasure within the *Anthropocene* [97]. Biochar is a C-rich solid formed by pyrolyzing biomass, and its direct addition to soil as such increases the C content. The preparation of biochar will partly fix the CO_2_ that plants absorb from the atmosphere through photosynthesis to charcoal, a more recalcitrant form of C [29]. Lehmann stated [29] that biochar offers the chance to turn bioenergy (generation) into a C-negative industry. Similarly to char-generation by fire and heat [97], hydrothermal carbonization (HTC) [98, 99] and hydrothermal humification (HTH) [27] are processes that can protect biomass-carbon from microbial degradation, here, even effectively wet biomass which is obviously hard to burn.

The physical addition of biochar is, however, just a single exogenous method and does not reduce the emission of CO_2_ from soil as such. On a second view, the chemical action of ACM on the soil can mitigate CO_2_ emissions to a large extent due to its abundant functionalities. A brief description of the most obvious reasons are as follows: (1) ACM applied in soil is able to increase nutrient availability for plant growth owing to its own nutrients (e.g., P, K, and elements) but also by promoting the soil to produce more usable nutrients. (2) It also will intensify photosynthetic efficiency resulting in the accumulation of C biomasses [100]; (3) ACM increases the water-binding capacity of soil, which is also a way of increasing productivity and thus fixing more atmospheric CO_2_ [101]. In comparison to the increase of the carbon content by physical and chemical actions of ACM in soil, we are more attracted by the potential of biological effects of ACM on soil to reduce or even invert C loss.

### 3.2. Carbon Regulates Biology

On top of these already powerful physicochemical effects, there are also potentially stronger biological amplification mechanisms. Inspired by the function of the soil microbiome within C accumulation, an anthropogenic regulated biological soil C pool seems to be a promising path. For that, the knowledge on how to regulate soil C flux by microbial respiration and metabolism, but also community building and structure, is vital, i.e., how and under which chemical conditions we can amplify the C sequestration ability of microbes or weaken their C emissions. Based on the current knowledge of the pathways of soil C fluxes related to microbiology, several hypothetical schemes can be identified.

#### 3.2.1. Metabolic Engineering Modified Microbe

Both autotrophic and heterotrophic microbes sequester C. This can be addressed with the advent of metabolic engineering [102, 103]. Hu et al. [102] emphasized several effective strategies to enhance the CO_2_ sequestration by autotrophic and heterotrophic microbes and reduce their CO_2_ emission via various advanced approaches. The enhancement of C sequestration efficiency can be carried out either by improving the natural metabolic pathways, e.g., by overexpressing CO_2_-fixing enzymes [104] and improving the catalytic properties of carboxylases [105] or designing new and more effective metabolic pathways, like a crotonyl-CoA/ethylmalonyl-CoA/hydroxybutyryl-CoA path [106], the PyrS-PyrC-glyoxylate cycle [107], and more. The development of technologies to reduce microbial CO_2_ emissions through designed metabolic pathways is also relatively mature. For instance, the introduction of nonoxidative glycolysis (NOG) path into *Escherichia coli* allows to convert C in the sugar catabolism to acetyl-CoA completely, while avoiding the emission of CO_2_ due to the decarboxylation of pyruvate in the natural aerobic oxidation path [108]. Overall, metabolic engineering modified microbes can be regarded as potentially effective to mitigate global climate change. However, the performance of such manipulated organisms in real soil is still to be analyzed, and their sustainability and competitiveness with the natural polytype and potential side effects, especially in arable soils, are to be most carefully examined.

#### 3.2.2. Artificial Carbon Materials Advance the Microbial Carbon Sequestration Potential

Compared to metabolic engineering, the performance of ACM in terms of biological regulation and influence on microbial health and diversity created a giant wave of scientific interest. Early investigations only focused on the stability of the soil C pool as well as on physical and chemical effects of ACM, but now, the effects of typical ACMs on the regulation of the microbial community and metabolism were found crucial.

As mentioned earlier, biochar as such is a rather stable, solid C structure, but it still affects microbes. In general, the growth of microbes benefits from a suitable soil habitat provided by biochar being responsible for its function of promoting soil nutrient availability buffering capacity and detoxification due to its specific structure and properties [23, 109]. However, an increment of microbe biomass does not necessarily increase C sequestration, as the balance between C loss and C increment by microbial biomass is to be assessed. Accordingly, biochar-induced changes or even inhibition of heterotrophic respiration of microbes, changes of the community structure and microbial abundance are key parameters to be analyzed. Some investigations revealed that the biochar amendment on soil reduced soil heterotrophic respiration strikingly resulting in a decreased C degrading microbial activity [110]. On the other side, Steinbeiss et.al [97] investigated that yeast-derived and glucose-derived biochar exposure to soil increased the respiration rate of soil microbes. Xu et.al [111] analyzed the reasons for the diversity of reactions of soil's heterotrophic respiration to biochar and found that biochar properties and amounts, climate conditions, exposure methods, and time make a difference. In another detailed study, the regulation of microbe community structure by biochar was analyzed. A higher addition of biochar to paddy soil pronouncedly increased the abundance of C sequestration genes, e.g., *cbbL* and *cbbM*, *accC*, and *hcd*, involved in Calvin-Benson cycle, 3-hydroxypropionate cycle, and 4-hydroxybutyrate cycle, separately, as identified by quantitative real-time polymerase chain reaction (*q*PCR) [112]. Redundancy analysis revealed that the redox potential, the C/N ratio of input fertilizer, and NO_3_^−^-N content had significant influence on the abundance of CO_2_ sequestering microbes.

Alteration of bacterial community structure depending on biochar amendment can improve C sequestration in soil. Ye et al. [113] described the variation of the diversity between bacterial communities on the surfaces of one type of biochar and two different mineral-enriched biochars after long-term incubation (140 d) in soil. Chemolithotrophic bacteria with the capacity of sequestrating additional CO_2_ were found dominant upon the surface of biochar.

Carbon sequestration efficiency of microbes can also be improved by stimulating expression and activity of specific enzymes. The abovementioned study [110] has already validated that the addition of biochar did increase Rubisco activity, the most important enzyme to control and mark microbial CO_2_ sequestration. CH_4_ emissions can be lowered by the exposure of biochar, too. For example, Wang et al. [114] proved that biochar amendment on paddy soils do reduce the emission of CH_4_ in a four-year study. Further, he clarified the role of the microbial community structure variation on the reduction of CH_4_ emission. Specifically, the application of biochar on paddy soils significantly suppressed the abundance of methanogens, while having less impact on the abundance and activity of methanotrophs.

Natural humic substances, which are widely present in surface soil, are most beneficial for microbial colonization, due to their ability to maintain a hydrated, pH, and redox buffered environment, and promote the release of abundant nutrients [27]. A-HS synthesis is new and thereby less analyzed. There are, however, some studies on how natural HS inhibits CH_4_ emissions from rice paddy soils, peatland soils, or wetland. Keller et.al [115] mentioned that HS can inhibit CH_4_ emission in wetlands, supported by its role of a thermodynamically favorable organic terminal electron acceptor (TEAs). In detail, there is a fierce competition between microbial CH_4_ generation and respiration under anaerobic conditions [116]. In general, microorganisms prefer to accept electrons from microbial oxidation of organic substrates (acetate and hydrogen), when there is sufficient HS in the soil [117]. This phenomenon is also found in peatlands. An investigation done by Ye et al. [118] claimed that the existence of HS acting as TEAs for anaerobic respiration in fen soils directly inhibits the emissions of CH_4_. Although the phenomenon that HS inhibits methane emissions is common to all wetlands, it is potentially due to different effects on methanogens [116]. The attached study in the work done by Cervantes et al. [116] claimed that the introduction of anthraquinone-2,6-disulfonate, a chemical redox buffer, and thereby an oversimplified model of HS, in the methanol-methanogen system, successfully delayed the CH_4_ production cycle and reduced the CH_4_ production by inhibiting acetoclastic methanogenic. Similarly, the investigation of Ye et al. [118] displayed that HS exposure to bog soils inhibited the production of CH_4_ by 86% owing to its inhibitive action on methanogens. Undeniably, these actions can contribute to the mitigation of the greenhouse effect because CH_4_ has a much bigger impact on the climate than CO_2_ [119].

**Figure 3:**
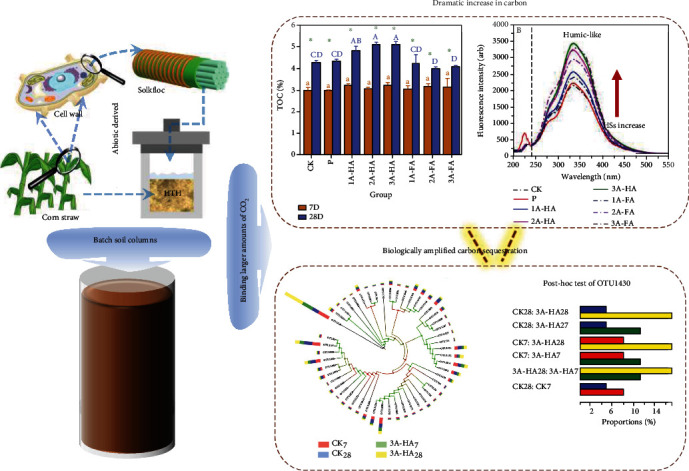
Artificial humic substances improve microbial activity for binding CO_2_.

HS is potentially able to accumulate other forms of C in soil, as HS holds much more functionality than biochar. At present, we have investigated that A-HS produced by hydrothermal humification technology (HTH) [38, 120, 121] substantially improved the content of SOC, whether under natural conditions or in a freeze-thaw environment [89, 122]. In one of our most recent studies [122] (Figure 3), we added up to 0.03 wt% C of an artificial humic or fulvic acid (A-HA and A-FA) to soil for improving soil texture and analyzed their influence on typical soil parameters of an already strong agricultural black soil from Harbin/China. Instead of partial metabolization and degradation, we found the opposite. As shown by the data, total organic carbon content of the soil increased by up to 2.1 wt% (compared to the added 0.03 wt% C). We could show that this increase was proportional to A-HA addition and the light shone on the soil sample. Genomic analysis of the bacteria growth in the soil allowed to identify *Rubrivivax gelatinosus* (a photosynthetic rhodobacterium, as well as a carboxydotrophic bacteria which can oxidize CO and fix CO_2_ even further through the Calvin-Benson cycle [77]). Rubrivivax gelatinosus is a key player in C sequestration, and the addition of A-HA obviously supports a rich *Rubrivivax gelatinosus* community. Calculating this effect to the scale of all farmed land, such an A-HA addition plus the induced biological amplification could make most countries C neutral. We underline that the addition of A-HA improves food productivity and phosphate activation at the same time.

**Figure 4:**
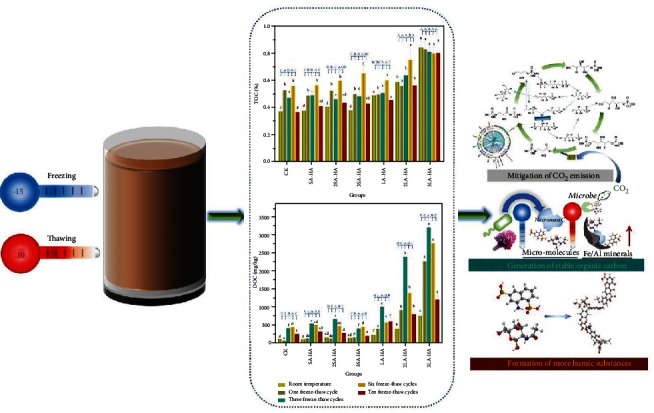
Artificial humic acid biologically amplifies carbon sequestration under freeze/thaw events [89].

Another investigation, which addressed the behavior of soil in cold temperature zones, analyzed the variation of SOC content with solid A-HA or A-HA solution amendment under freezing-thawing alternation [89] (Figure 4). The longest experiment cycle was 10 freeze-thaw cycles (one freeze-thaw cycle is -15°C for 2 days and 10°C for 5 days). The resulting data shows that the addition of A-HA solution significantly increased the stable carbon pool in soil. Additionally, the experimental conditions in this case excluded the influence of photosynthetic bacteria on soil C sequestration. One can thereby deduce that the bacteria taking part were mainly aerobic chemoautotrophic bacteria. Results of high-throughput sequencing demonstrate that the addition of a special amount of A-HA solution significantly altered the structure of soil sequestration carbon bacteria communities, especially decreasing the relative abundance of *Cupriavidus necator*, a nonobligate bacterial predator, and promoting the relative abundance of *Mycobacterium gadium* and *Aurantimonas manganoxydans*. Meanwhile, the addition of A-HA solution also critically stimulated bacterial growth as revealed by the results of qPCR results.

Concluding, the increased carbon pool depends on a series of combined triggers:

1. Increased stable carbon is in the form of a combination of microbial necromass and Fe/Al mineral complexes
2. A-HS, with no doubt, is capable of promoting microbe growth, creating excellent habitats for soil microbes and providing available nutrients [89, 95]
3. A-HS functions to inhibiting the activity of extracellular enzymes [123]. Notable, the phenomenon of “locking” carbon in freezing-thawing events is intuitively clear owing to the dual inhibition of extracellular enzymes by A-HS and freezing actions. Another theory, denoted as mineral “latch,” is another effective path to lock soil carbon [124, 125]. The Fe “latch” is expected to work better under aerobic conditions when A-HS is present as its redox buffer capacity can promote the reduction efficiency of iron-reducing microorganisms to reduce ferric iron to ferrous iron
4. A-HA plays a central role in regulating carbon sequestration bacterial community structure to amplify carbon sequestration efficiency and organizing cascades and exchange by the “currency” of microbial patches that are buffered electron-proton pairs
5. The degradation of dissolved fulvic acids (essentially potentially labile organic carbon sources) and weaker humic acids effectively generates more stable humic substances

These views should be taken as a working hypothesis to tackle the critical questions of artificial humic matter in soil. In general, natural humic substance is a relatively resistant material in soil with an apparent turnover rate of approximately 50 yr, more resistant than resistant plant detritus (3.5 yr) or decomposable plant material (1 month) [126], and Swift [126] proposed that the further decomposition of humified organic matter will create even more humified organic matter, which is due to a complex biological cascade. In our investigation [89], we could uncover only some key operations of A-HS in the soil. For example, UV-vis spectra results show that the addition of A-HA solution introduced natural chromophore formation (e.g., quinone and polyphenols) bound to auxochromes (e.g., carboxyl and hydroxyl) and thereby indicated enhanced humification. In addition, the addition of A-HA solution increased the hydrophobicity, which also strengthens the resistance to bacterial degradation.

## 4. The Bigger Picture

Global warming receives urgent attention by humankind and should be considered by all scientists, even if only as a side activity in the own topic. The most basic climate models suggest that if the amount of CO_2_ in the atmosphere doubles from preindustrial levels (to 560 ppm), the planet warms by between 1.5 and 4.5°C in 2100 [127]. The newest climate model presented by 25 scientists from the World Climate Research Program (WCRP) further narrowed in 2020 the climate sensitivity range to a scale of 2.6 and 3.9°C, and this prediction was rated as one of the top 10 scientific breakthroughs by *Science*. The study ruled out some worst-case scenarios, but it confirmed that global warming would inundate coastal cities, exacerbates extreme heat waves, and displace millions of people. Not only the latest investigation demonstrated that the climate change is faster than thought before reaching the threshold as early as 2060, accordingly, action on climate change by inventing carbon negative products and processes is urgently needed, at best with a scalable simple technology bringing a high (biological) system lever. Research and application of carbon to soil is such a well described action, and for instance, set the base on the so-called “4 per 1000” initiative of the *Paris Climate Convention* [128].

Conversely, investigation and applications of HS and especially artificial humic substances (A-HS) to soil for C sequestration are still rare; although, it is a naturally occurring carbon carrier in soil. Recent investigations have revealed that A-HS (as a human-made humus analogue) greatly alters the interplay and community structure of soil C sequestration bacteria and thereby comes with an enormous biological amplification factor. *Rubrivivax gelatinosus*, which was identified to undergo the most obvious changes, does not only fix CO_2_ by photosynthesis directly but is an early earth universalist, that can activate a series of carbon conversion methodologies. Microbe necromass is then the major contributor to C accumulation in soil; yet, its variation induced by artificial soil carbon materials is less reported. Promotion of microbial growth by artificial carbon materials is meanwhile well proven; however, the turnover of the microbial community still needs further testing to confirm the general value. Foreseeably, the development of advanced artificial carbon materials, especially A-HS synthetized from omnipresent side stream biomass, has all the chances to contribute to a global carbon balance and management and maintain the fertility and agricultural productivity of farming soils.
